# Challenges and advances in drug resistance and tolerance in cancer

**DOI:** 10.1186/s13046-026-03665-y

**Published:** 2026-03-04

**Authors:** Trever G Bivona, Itai Yanai, Fabio Calabrò, Fatima Mechta-Grigoriou, Giacomo Corleone, Marta Di Martile, Annalisa Tocci, Piera Tocci, Sebastiano Vaccarella, Marilia Consiglia Ferriero, Andrea Alimonti, Eytan Ruppin, Paola Nisticò, Anna Bagnato, Giovanni Blandino

**Affiliations:** 1 Department of Medicine, University of California, San Francisco, USA; 2 NYU Langone Health, New York, USA; 3 Medical Oncology 1, IRCCS Regina Elena National Cancer Institute, Rome, Italy; 4 Stress and Cancer Laboratory, Institut Curie, Paris, France; 5 Gene Expression and Cancer Model Unit, IRCCS Regina Elena National Cancer Institute, Rome, Italy; 6 Preclinical Models and New Therapeutic Agents Unit, IRCCS Regina Elena National Cancer Institute, Rome, Italy; 7 Tumor Immunology and Immunotherapy Unit, IRCCS Regina Elena National Cancer Institute, Rome, Italy; 8 Translational Oncology Research Unit, IRCCS Regina Elena National Cancer Institute, Rome, Italy; 9 Department of Urology, IRCCS Regina Elena National Cancer Institute, Rome, Italy; 10 Department of Molecular Oncology, Institute of Oncology Research, Bellinzona, Switzerland; 11 Data Science Laboratory, Center for Cancer Research, National Cancer Institute, Bethesda, USA; 12 Scientific Direction, IRCCS Regina Elena National Cancer Institute, Rome, Italy

**Keywords:** Drug resistance, Translational oncology, Precision oncology, Immunotherapy

## Abstract

Therapeutic resistance remains the principal barrier to durable clinical benefit in oncology, particularly in oncogene-driven malignancies and immune-refractory tumors. This meeting brought together leading experts to dissect the multifaceted biological mechanisms underlying drug tolerance, adaptive resistance, and immune escape across diverse cancer types. Presentations highlighted how cancer cell-intrinsic plasticity, chromatin reprogramming, and stress-responsive transcriptional networks intersect with tumor microenvironment-derived cues, including inflammatory signaling, stromal heterogeneity, mechanotransduction, and paracrine crosstalk, to sustain drug-tolerant persister states. Novel insights into cancer-associated fibroblast plasticity, spatially organized immunosuppressive niches, secretory autophagy, and senescence-associated programs underscored the dynamic and adaptive nature of resistance. Cutting-edge approaches, including single-cell and spatial transcriptomics, chromatin accessibility profiling, organoid-based co-culture platforms, and artificial intelligence-driven spatial inference, revealed actionable vulnerabilities and predictive biomarkers. Collectively, these studies emphasize that resistance is not a binary phenomenon but a continuum shaped by evolutionary adaptation and ecological interactions within the TME. This report synthesizes the conceptual advances and translational implications emerging from the meeting, outlining new therapeutic strategies aimed at disrupting adaptive tolerance states, reprogramming immunosuppressive niches, and enabling fast, accessible precision oncology.

## Introduction

The IRE Workshop on Translational Oncology is an annual international conference organized by the IRCCS Regina Elena National Cancer Institute of Rome, Italy, in collaboration with leading research institutions worldwide. Its mission is to foster in-depth discussion of emerging scientific discoveries with the highest potential to transform precision oncology, bridging fundamental research with clinical innovation.

The 7th Annual IRE Workshop, held in 2025, was dedicated to the theme “Challenges and advances in drug resistance and tolerance in cancer” and was organized in collaboration with the Weizmann Institute of Science (Rehovot, Israel) and the National Institutes of Health, National Cancer Institute (Bethesda, USA). The meeting took place at the Regina Elena National Cancer Institute Conference Center in Rome, Italy, from October 20–21, 2025.

The workshop gathered approximately 200 investigators, with early-career researchers representing nearly half of the participants, underscoring the strong commitment to training and scientific renewal in the field. The scientific program comprised five thematic sessions encompassing 22 invited and selected talks, addressing resistance and tolerance through complementary perspectives, including genomic and epigenetic regulation, tumor microenvironment dynamics, mechanobiology, and systems biology approaches.

This meeting report summarizes the key contributions of the 7th IRE Workshop, highlighting cutting-edge advances in the understanding of cellular adaptation to anticancer therapies. By disseminating these insights, we aim to stimulate new research directions, promote interdisciplinary collaboration, and accelerate the development of more effective therapeutic strategies capable of overcoming resistance and ultimately improving patient outcomes. The report collected some presentations presented during the Workshop.

### Insights into multifaceted mechanisms underlying drug tolerance and resistance in oncogene-driven NSCLC

#### *Trever Bivona* (University of California, San Francisco, USA)

**Fig. 1:**
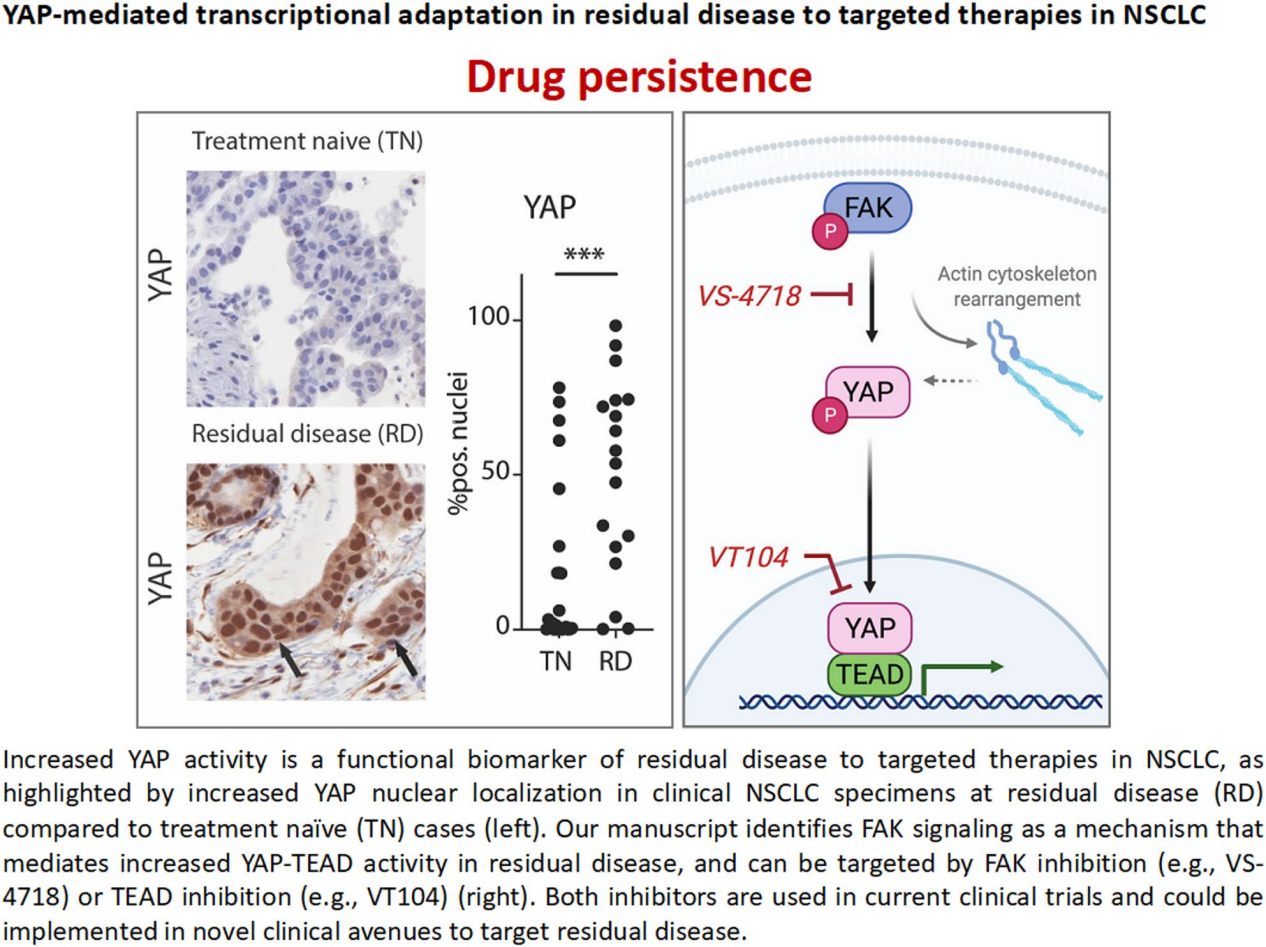
Cancer therapy persistence limits clinical responses in many cancers including lung cancer. One pathway described by many groups that mediates persistence is the YAP/TEAD (Hippo) pathway, shown below and described in Refs. 2 & 3 above and left panel below in clinical MRD samples. Blocking this pathway with inhibitors of YAP/TEAD or the upstream activating kinase FAK can overcome this persistence in preclinical models, as shown in the right panel below

Despite important progress, lung cancer remains the leading cause of cancer mortality worldwide. A major barrier to improving long-term survival outcomes for lung cancer patients is the problem of cancer therapy resistance. One form of this resistance is drug tolerance, which manifests during treatment as a reservoir of drug tolerance persister cancer cells that survive therapy by both cancer cell intrinsic and extrinsic mechanisms across the tumor-tumor microenvironment. The work discussed in my presentation showed several multi-dimensional mechanisms of tolerance to targeted therapy in lung cancer as revealed by dual clinical specimen and experimental model analyses. Key mechanisms include the Hippo-YAP pathway and WNT/Beta-catenin signaling as well as inflammatory processes such as NFkB pathway activation and a tumor macrophage cytokine circuit that promoted tolerance to EGFR and KRAS inhibitors. The dataset contains key targets for clinical translation in the form of clinical combination therapy trials in biomarker selected lung cancer patients, some of which are now ongoing [1–4]. (Figure 1).

### Cellular adaptation to cancer therapy and the tumor microenvironment

#### *Itai Yanai* (New York University, New York, USA)

**Fig. 2:**
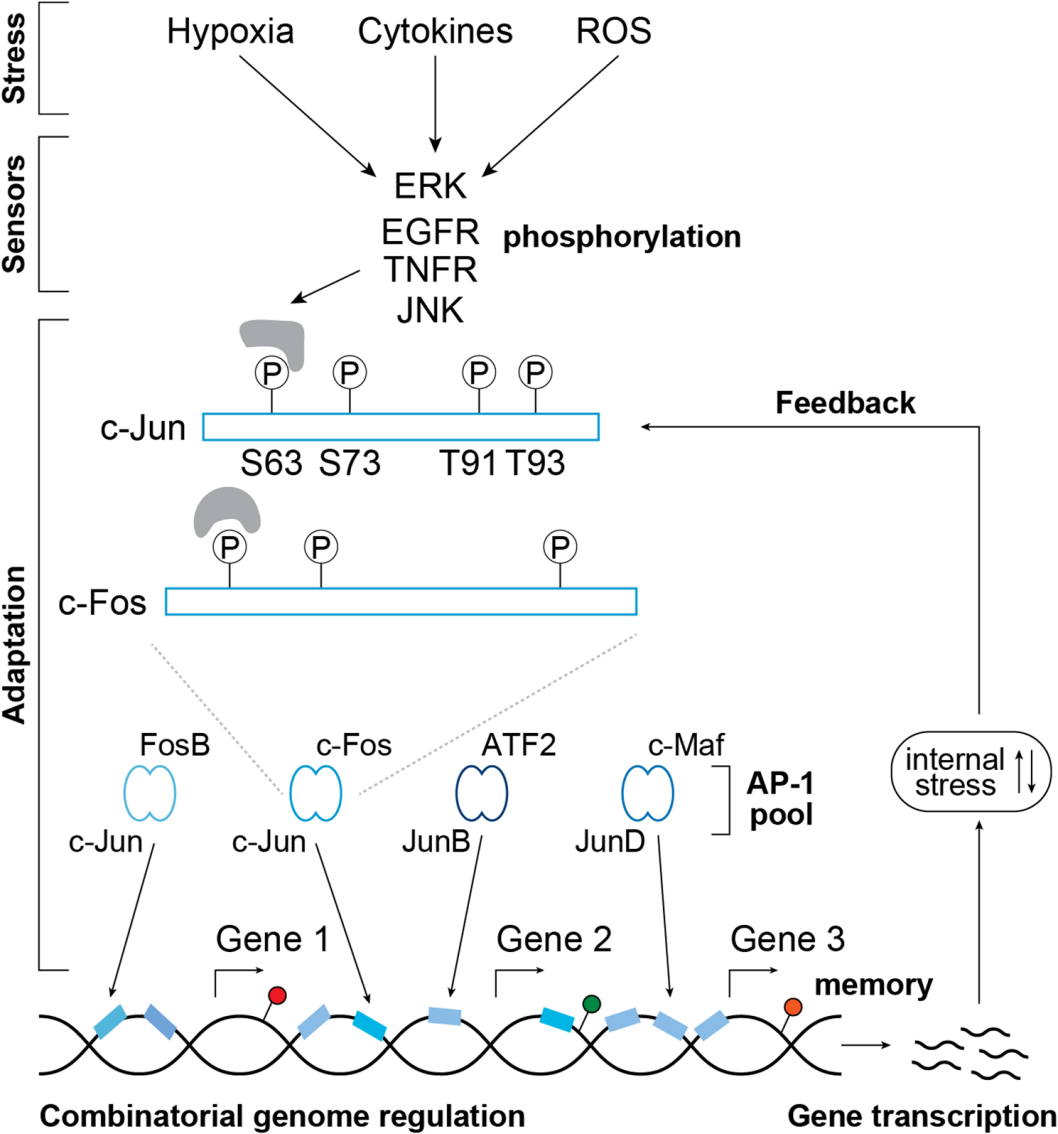
A model for cellular adaptation to stress

We previously introduced the concept of the ‘resistance continuum’, in which drug resistance emerges through progressive adaptive cell-state transitions toward increased fitness, underpinned by phenotypic plasticity and chromatin reprogramming for stress response [5]. In my talk, I presented a model that Gustavo S. França and I have developed on combinatorial genome regulation through the AP-1 system [6]. The model involves the integration of diverse environmental signals sensed by molecular sensors that converge on AP-1 activation (see Figure). Once activated, AP-1 dimers bind to widespread regulatory elements across the genome to modulate transcription. This exploratory mode of gene regulation leads to the expression of genes that can either mitigate or exacerbate internal stress, which further feeds back into the AP-1 system. In this model, the tuning of optimal responses depends on the regulated expression of AP-1 subunits, which constitutes the pool of dimer compositions and determines target gene selection and engagement with the broad regulatory landscape (blue squares). Finally, I highlighted the broader potential relevance of these mechanisms – extending beyond cancer to contexts such as inflammation and neurobiology – and the insights they offer into the origin and evolution of novel gene regulatory programs. (Figure 2).

### Immunotherapy in urothelial cancer

#### *Fabio Calabrò* (IRCCS Regina Elena National Cancer Institute, Rome, Italy)

**Fig. 3:**
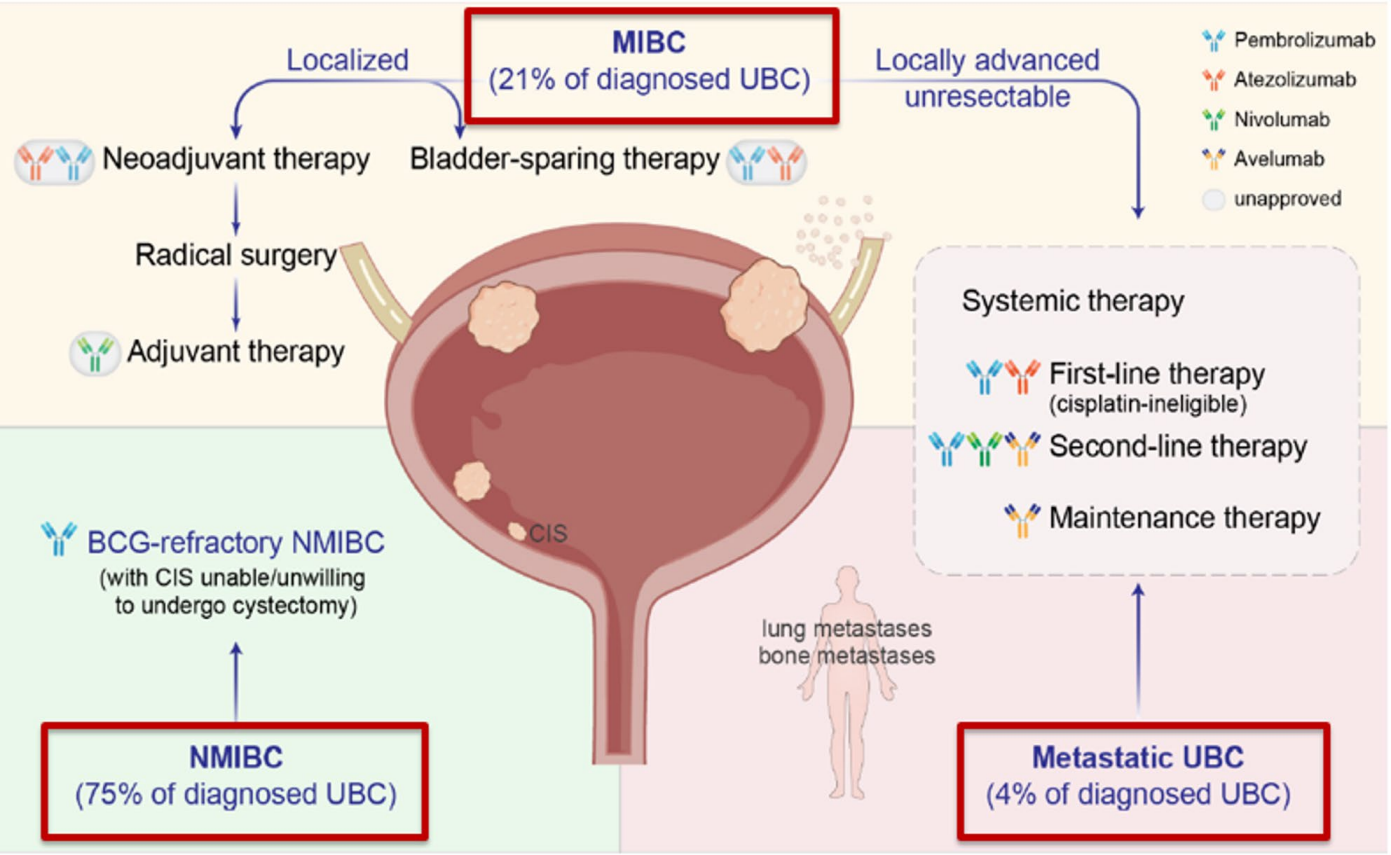
Urothelial carcinoma of the bladder. Many different diseases and treatments

The rationale for immunotherapy in urothelial carcinoma is very solid. BCG has been used since the 1990s to reduce the risk of recurrence in non-muscle-infiltrating bladder carcinoma. In this area, many new immunotherapeutics have already been registered by the FDA. Some of these use viral vectors, while others are systemically administered. In muscle-infiltrating disease, several studies with checkpoint inhibitors after cystectomy have not yet demonstrated a clear survival advantage, while data on the use of immunotherapy in combination with chemotherapy in the pre-operative treatment of muscle infiltrating bladder cancer have clearly shown a survival advantage. Even in metastatic disease, immunotherapy in combination with antibody drug conjugates has been shown to double the median survival time as compared to standard chemotherapy regimens used in the last 25 years. The future of urothelial carcinoma treatment therefore looks bright and immunotherapy will certainly be the cornerstone of treatment in the coming years. Routine use of ctDNA for disease monitoring and the discovery of novel predictive biomarkers of response will be appropriate to improve the results achieved to date [7–10]. (Figure 3).

### Deciphering Spatial heterogeneity of the stroma and immunosuppression in breast cancer

#### *Fatima Mechta-Grigoriou* (Institut Curie, Paris, France)

**Fig. 4:**
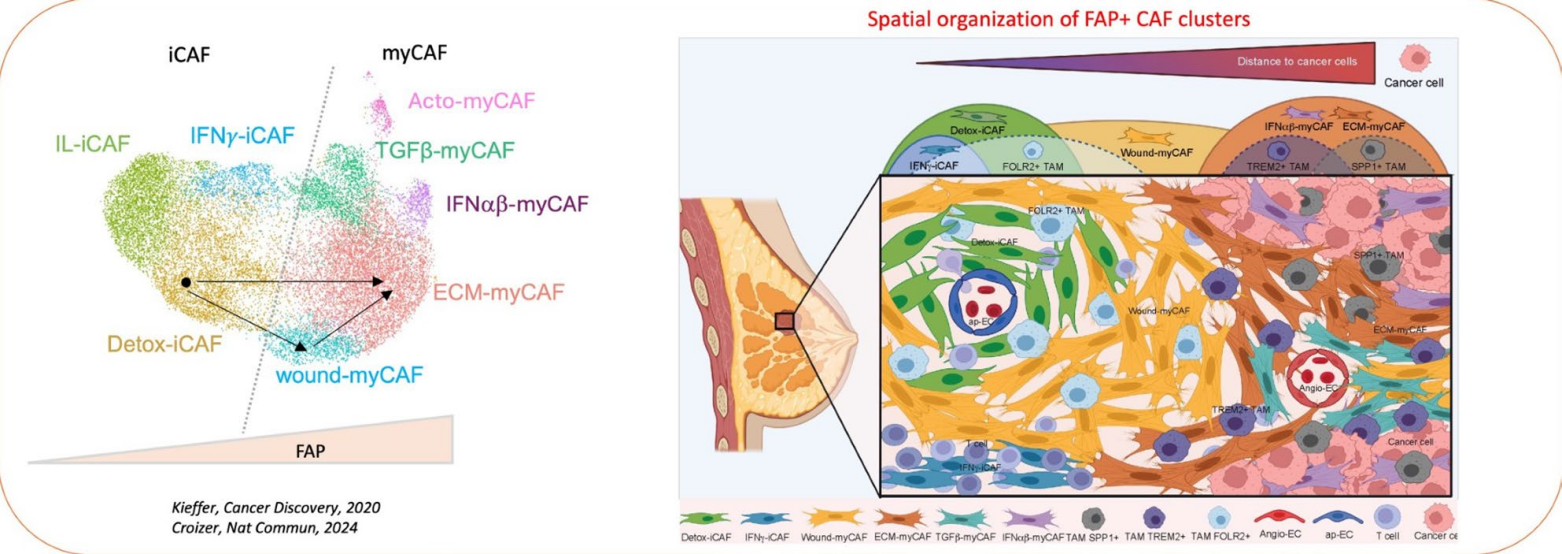
CAF heterogeneity and plasticity shape a structured organization of the tumor microenvironment in BC. The analysis of the FAP + CAF in different pathological conditions showed that they could be divided into two main categories, according to their expression of ANTXR1 and FAP: iCAF (Detox-, IL-, IFNγ-iCAF) are negative for ANTXR1 expression, and express low levels of FAP, whereas myCAF (Wound-, ECM-, TGFβ-, Acto-, IFNαβ-myCAF) are positive for ANTXR1 expression, and express higher levels of FAP. In the context of BC, we showed that distance to cancer cells induces a gradient of FAP + CAF cluster identities, which participate to shape distinct spatially-organized cellular niches. Detox-iCAF are found around blood vessels composed of antigen-presenting endothelial cells (ap-EC) and are in close proximity to FOLR2 + tumor-associated macrophages (TAM). Detox-iCAF serve as a reservoir and can give rise to ECM-myCAF in presence of cancer cells, either directly or indirectly through the Wound-myCAF cluster, by DPP4-and YAP1/TEAD-dependent mechanisms. ECM-myCAF localize close to tumor cells, where they can reach a TGFβ-myCAF phenotype in presence of T lymphocytes. In addition, specific TAM are found in different FAP + CAF cluster-enriched territories. While FOLR2 + TAM are close to Detox-iCAF, TREM2 + and SPP1 + TAM are enriched in ECM-myCAF, IFNαβ-myCAF and TGFβ-myCAF-enriched niches

Our work revealed Cancer-associated fibroblasts (CAF) heterogeneity and showed their critical roles in cancer. We identified different CAF populations, including myofibroblastic FAP + CAF and FAP- pericyte-like fibroblasts [11], which display a pro-metastatic role [12]. FAP + CAF also exhibit immunosuppressive functions [11]. Highly resolutive single cell analysis of FAP + CAF identified 3 inflammatory ANTXR1- iCAF clusters and 5 myofibroblastic ANTXR1 + myCAF clusters [13]. These FAP+ ANTXR1 + myCAF clusters are immunosuppressive and associated with resistance to chemo- and immunotherapies [13, 14]. Recently, we highlighted 10 spatially-organized FAP + CAF cluster-related niches [15]. Cancer cells drive the transition of detoxification-associated iCAF (Detox-iCAF) towards immunosuppressive ECM-producing myCAF (ECM-myCAF) via a DPP4- and YAP-dependent mechanisms. In turn, ECM-myCAF polarize immunosuppressive niches (enriched in TREM2 + macrophages and regulatory T cells), while Detox-iCAF are associated with immunoprotective niches (enriched in FOLR2 + macrophages). FAP + CAF clusters accumulate differently according to the invasive cancer status and predict invasive recurrence of breast cancer. The existence of these clusters is validated in various cancer types, as well as in chronic kidney disease [16], suggesting broad pathological relevance. (Figure 4).

### Chromatin profiling identifies actionable vulnerabilities in resistant multiple myeloma

#### *Giacomo Corleone* (IRCCS Regina Elena National Cancer Institute, Rome, Italy)

**Fig. 5:**
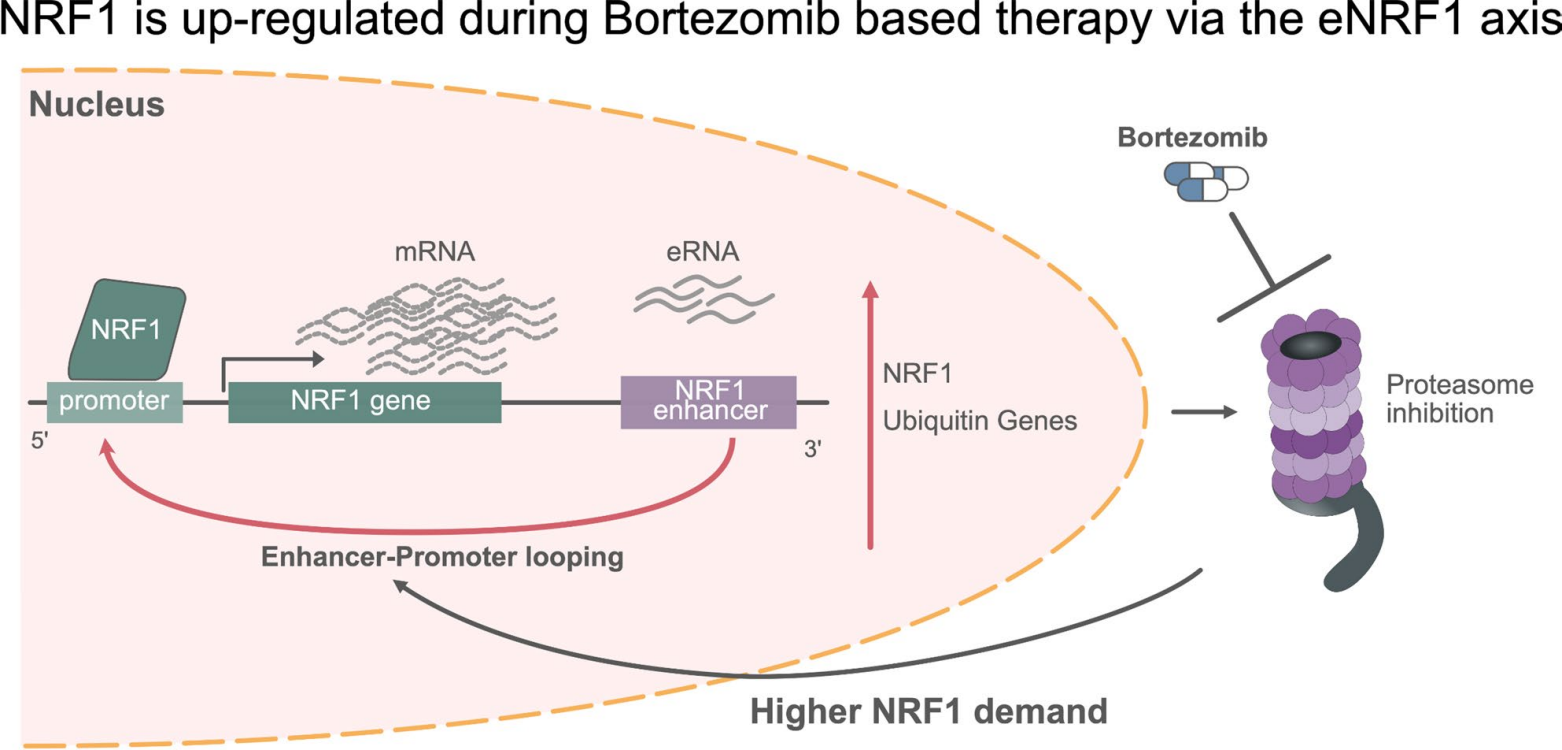
NRF1 is up-regulated during Bortezomib based therapy via the eNRF1 axis

Although the efficacy of current treatments is limited, 40% of Multiple Myeloma (MM) patients relapse within 10 years of diagnosis. This highlights the urgent need for innovative therapy approaches. In this work, Bruno, Cappelletto, & Cortile et al. [17] used ATAC-seq to profile chromatin accessibility in 55 patients. They identified that the ubiquitous [18] protein Nuclear Respiratory Factor 1 (NRF1) regulates essential myeloma cell survival pathways, regardless of cytogenetic group. Specifically, NRF1 maintains proteasome homeostasis by orchestrating the critical ubiquitin-proteasome pathway. Under bortezomib treatment, primary resistant myeloma cells upregulate NRF1 as an adaptive survival mechanism. This activation is driven by a previously unknown RNA-productive enhancer element positioned 170 kb downstream of the NRF1 promoter. Importantly, inhibiting the NRF1 enhancer using antisense oligonucleotides (ASO) reduced NRF1 levels and sensitized cells to bortezomib in vitro and in vivo. Collectively, the NRF1-enhancer axis represents a promising therapeutic target to enhance bortezomib efficacy and overcome resistance in MM. (Figure 5).

### Targeting the Bcl-2 regulatory network to address immunotherapy resistance in preclinical melanoma models

#### *Marta Di Martile* (IRCCS Regina Elena National Cancer Institute, Rome, Italy)

**Fig. 6:**
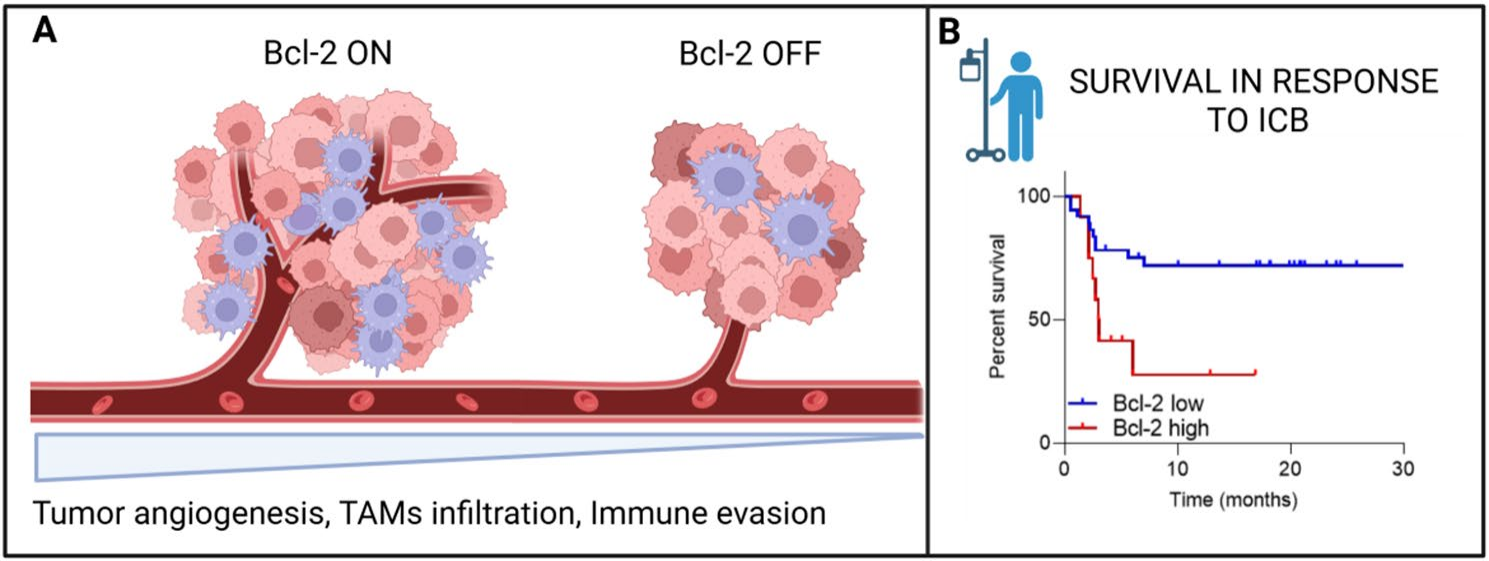
**A **Schematic representation of the scenario in the presence (left panel) or absence (right panel) of Bcl-2 protein in melanoma tumors. Blue cells: TAMs; Brown cells: tumor cells. **B **Survival curves from https://kmplot.com for melanoma patients with high or low Bcl-2 protein levels treated with anti-PD-1 or anti-CTLA4 monoclonal antibodies. Abbreviations: TAMs, Tumor-Associated Macrophages; ICB, Immune Checkpoint blockade. *Figure created with Biorender.com*

The development of new therapeutic combinations addressing the resistance to immune checkpoint blockade (ICB) therapy is a clinical need to improve the management of metastatic melanoma patients. In this scenario, the anti-apoptotic protein Bcl-2 impaired the response to chemo- and target- therapy and promoted melanoma progression and aggressiveness [19–21]. Importantly, we demonstrated that melanoma-specific Bcl-2 boosts some mechanisms fueling the ICB resistance, such as tumor angiogenesis [19] and the recruitment of immune suppressive cells including pro-tumoral macrophages [22] (Fig. 6A). Furthermore, we evidenced that Bcl-2 expression is associated with a poor clinical outcome of melanoma patients treated with anti-PD-1 or anti-CTLA4 monoclonal antibodies ICB therapy (Fig. 6B). Our data pave the way for the identification of a new Bcl-2-based therapeutic strategy to counteract the ICB resistance in advanced melanoma.

### hMENA-regulated secretory autophagy mediates unconventional communication between CAFs and cancer cells contributing to immunotherapy resistance in NSCLC

#### *Annalisa Tocci* (IRCCS Regina Elena National Cancer Institute, Rome, Italy)

A deeper understanding of noncanonical paracrine interactions between cancer-associated fibroblasts (CAFs) and tumor cells, is essential to identify new vulnerabilities in the tumor microenvironment (23). We explored how the actin-regulator hMENA and its isoform hMENAΔv6 (24) influence secretory autophagy in CAFs and, in turn, shape tumor cell behavior in non-small cell lung cancer (NSCLC). Modulating hMENA expression in primary CAFs affected their autophagy-related secretory activity and altered the release of protumoral factors, ultimately impacting tumor cell proliferation and metabolic adaptation.

At the clinical level, transcriptomic analyses revealed that a gene signature, linked to secretory autophagy and hMENA expression, is enriched in specific CAF subtypes associated with unfavourable patient outcomes. The construction of a single-cell CAF atlas and spatial profiling further highlighted the spatial proximity of these CAF populations to tumor regions in patients with poor responses to immune-checkpoint therapy (ICT). Integrative computational approaches showed that, combining this signature with CAF-state information, improves the identification of immunotherapy-nonresponsive tumors in independent NSCLC cohorts.

**Fig. 7:**
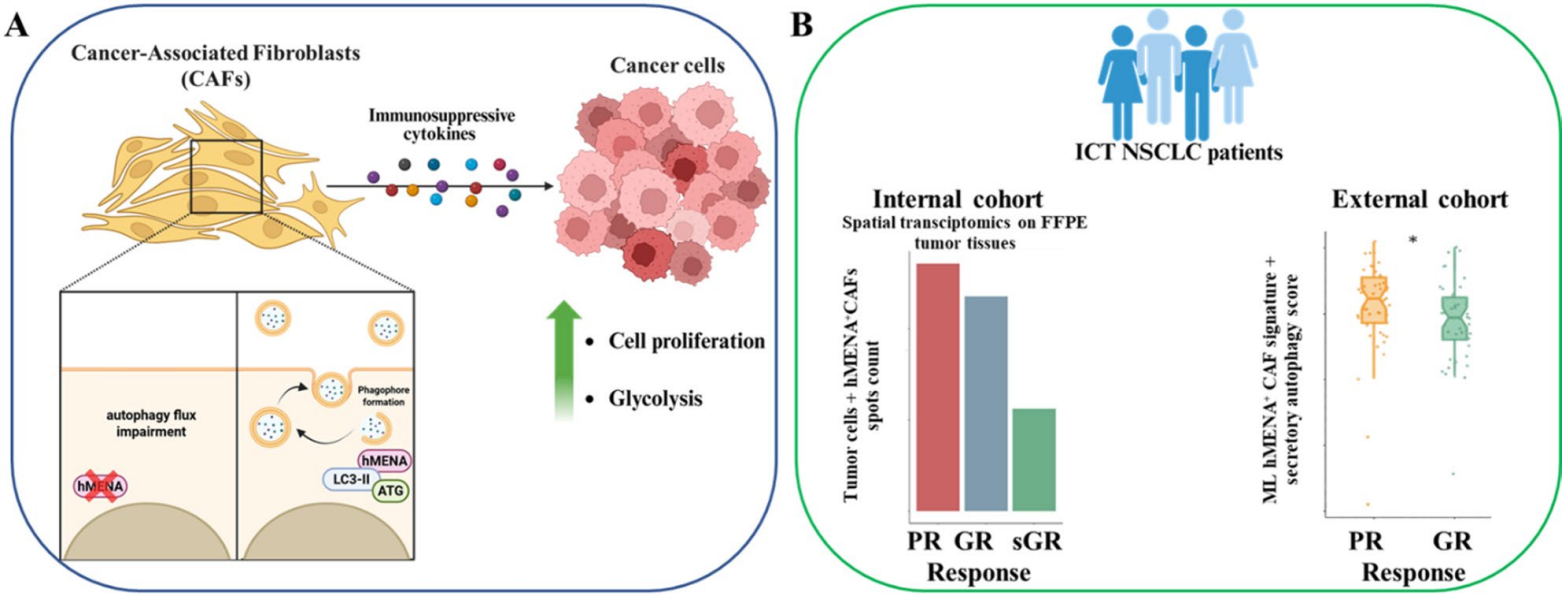
**A **hMENA promotes both autophagy and secretory autophagy in cancer-associated fibroblasts (CAFs), fostering cytokine/chemokine release that drives NSCLC cell growth and metabolic rewiring. **B **In poor-responder patients, spatial transcriptomics reveals a colocalization between tumor cells and hMENA⁺ CAFs secreting immunosuppressive cytokines. Machine-learning (ML) analyses link a secretory-autophagy signature together with hMENA⁺ CAF-derived immunosuppressive cytokines to reduced sensitivity to immune checkpoint therapy (ICT). PR= poor responders; GR= good responders; sGR= super good responders. *Figure created with Biorender.com*

Together, these findings point to a previously underappreciated role of hMENA-regulated secretory programs in CAF-tumor communication, suggesting a potential avenue for therapeutic intervention to overcome resistance to immunotherapy in NSCLC (Fig. 7).

### Targeting endothelin-1 receptor/PIEZO1 mechanosignaling to tackle drug resistance in high-grade serous ovarian cancer

#### *Piera Tocci* (IRCCS Regina Elena National Cancer Institute, Rome, Italy)

**Fig. 8:**
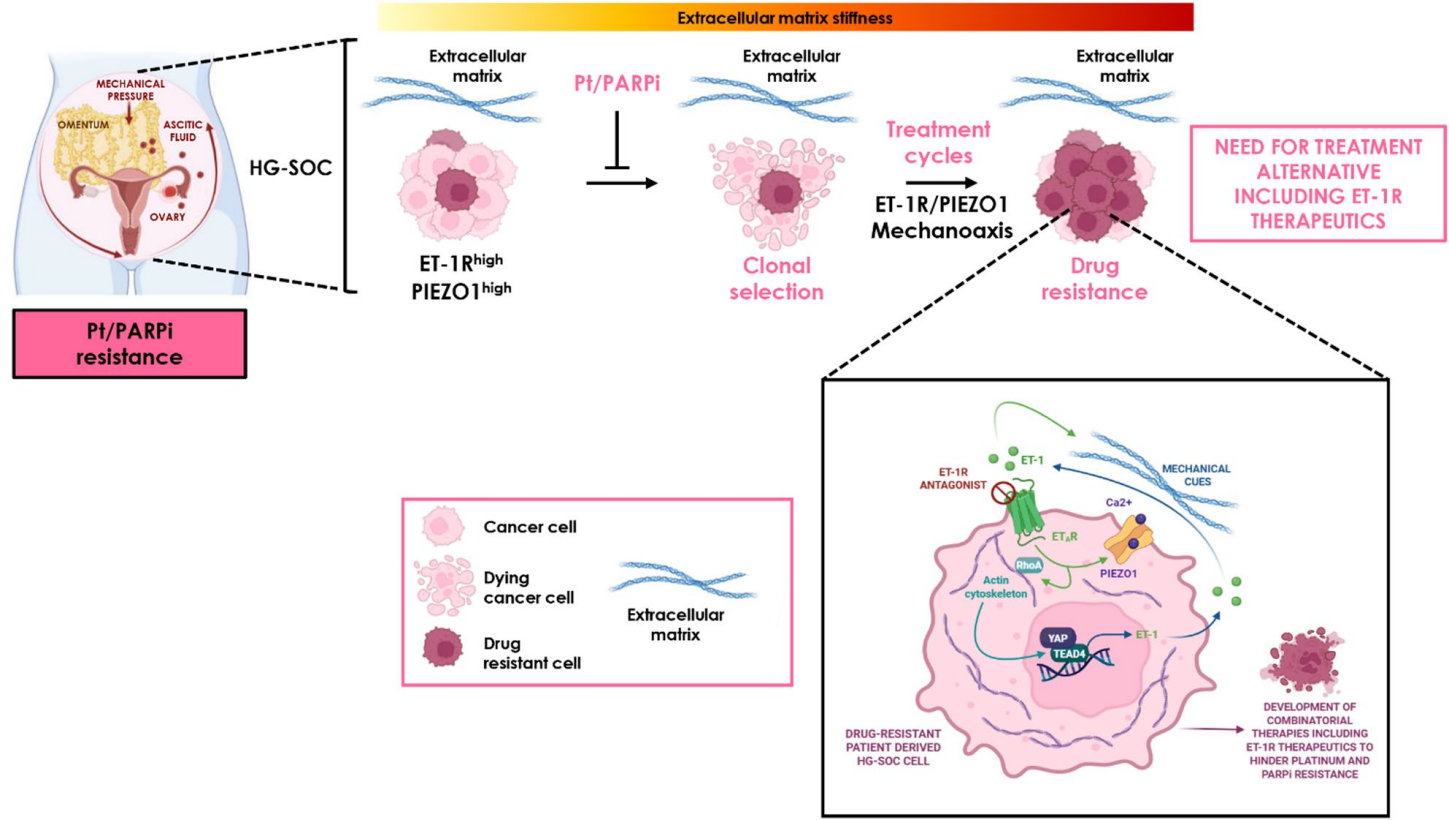
Schematic model illustrating how increasing extracellular matrix (ECM) stiffness in HG-SOC drives clonal selection of a Pt/PARPi-resistant phenotype. At molecular level, the stiffened-ECM-generated mechanical forces cooperate with the ET-1/ET-1R signaling and PIEZO1 to engage the YAP/TEAD4 transcriptional machinery, thereby sustaining tumor progression and fueling Pt/PARPi resistance. ET-1R blocking compounds disrupt this mechanocircuit, positioning ET-1R-targeting therapeutics as a powerful approach to overcome Pt/PARPi resistance and improve HG-SOC patient outcomes. *Figure created with biorender.com*

Platinum (Pt)-based chemotherapy and poly ADP-ribose polymerase (PARP) inhibitors (PARPi) resistance severely limit the high-grade serous ovarian cancer (HG-SOC) management. Beyond cancer cell-intrinsic mechanisms, growing evidence points to the tumor mechanics, particularly the extracellular matrix (ECM) stiffening, as a major contributor of treatment failure in HG-SOC [25]. We previously identified the endothelin-1 (ET-1)/ET-1 receptors (ET-1R) axis as a critical driver of tissue fibrosis and resistance to Pt and PARPi [26, 27]. Nevertheless, defining how ET-1 signaling in concert with dysregulated mechanical cues, orchestrates the acquisition of a resistant phenotype, would contribute to clarify the mechanisms underpinning drug resistance onset and enable the identification of therapeutic targets to improve patient survival prospects. In this study, using Pt- and PARPi-responsive and unresponsive patient-derived (PD) HG-SOC models, we uncover that the ET-1R axis and the mechanosensitive calcium channel PIEZO1 [28], through RhoA/F-actin signaling, activate YAP/TEAD4. The mechanocircuit between ET-1R/PIEZO1 and mechanoforces regulate Pt/PARPi therapy response. Pharmacologic ET-1R blockade with the ET-1R antagonist macitentan dismantles this network under stiff extracellular matrix (ECM)-derived forces, restoring Pt/PARPi sensitivity. In HG-SOC PDX, ET-1R therapeutics synergizes with PARPi suppressing the HG-SOC metastatic course. Notably, the ET-1R/PIEZO1/YAP gene signature emerges as an indicator of unfavourable therapeutic response and prognosis. These findings establish ET-1R inhibition as a compelling strategy to overcome Pt/PARPi resistance and guide future combination therapies in HG-SOC. (Figure 8).

### High-throughput drug screening on organoid platforms: targeting cancer associated fibroblast to overcome chemo-resistance in endometrial carcinoma

#### *Sebastiano Vaccarella* (IRCCS Regina Elena National Cancer Institute, Rome, Italy)

**Fig. 9:**
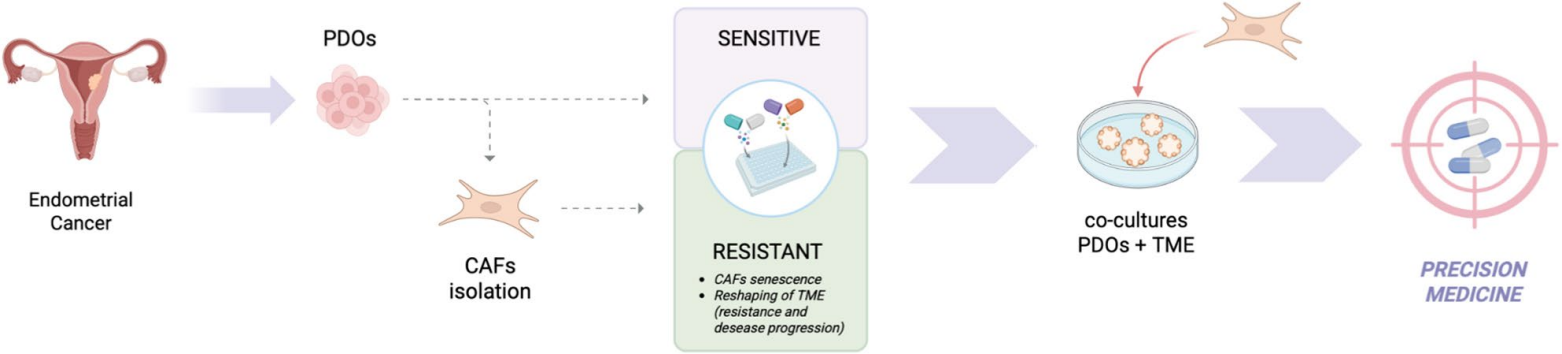
Endometrial cancer (EC) tissues are dissociated to generate patient-derived organoids (PDOs). Following PDO establishment and expansion, matched cancer-associated fibroblasts (CAFs) are isolated and characterized using genomic and molecular analyses. Once organoid and CAF cultures are established, drug screening is performed. Top candidate compounds are subsequently evaluated in PDO–CAF co-culture models to optimize precision medicine strategies

Patient-derived endometrial cancer organoids (EC-PDOs) have emerged as advanced 3D tumor models capable of faithfully recapitulating key histopathological, molecular, and functional features of the original tumors, providing a robust platform to investigate tumor-intrinsic drug responses and mechanisms of therapy resistance [29]. However, tumor behavior and treatment outcome are profoundly influenced by the tumor microenvironment (TME), in which cancer-associated fibroblasts (CAFs) play a central role [30]. Recent evidence indicates that CAFs not only promote aggressive tumor phenotypes but also display intrinsic resistance to therapy. In line with these observations, our data show that endometrial cancer (EC)-derived CAFs are significantly less sensitive to standard chemotherapy compared with matched EC-PDOs. We further demonstrate that CAF chemoresistance is associated with senescence-associated mechanisms, which we hypothesize may remodel the TME through paracrine signaling, thereby mediating drug resistance and promoting disease progression [29]. Although 3D tumor models are rapidly advancing, most are not yet ready for routine clinical application. Early-passage PDOs can capture key aspects of tumor complexity, while biobanking PDOs from treatment-naïve and therapy-resistant tumors provides a valuable platform to dissect resistance mechanisms and identify therapeutic strategies. As interventional studies integrating PDOs with TME-informed co-cultures progress, they will clarify the clinical impact of 3D preclinical models on cancer therapy [31]. (Figure 9).

### Drug resistance in genito-urinary malignancies: treatment options for BCG unresponsive bladder cancer and castration resistant prostate cancer

#### *Marilia Consiglia Ferriero* (IRCCS Regina Elena National Cancer Institute, Rome, Italy)

Drug resistance is a major obstacle in the management of genito-urinary malignancies. Despite advances in immunotherapy and targeted agents, many patients with bladder or prostate cancer experience disease progression due to adaptive resistance mechanisms.

In Non muscle invasive bladder cancer (NMIBC), up to 40% of patients fail BCG therapy, resulting in the BCG-unresponsive phenotype. Mechanisms include reduced immune cell recruitment, impaired cytokine signaling, and tumor-driven immune evasion. Radical cystectomy remains the gold standard, yet several bladder-preserving approaches show promise. These include intravesical chemotherapy combinations (gemcitabine/docetaxel), Immune checkpoint inhibition and gene therapy vectors.

In prostate cancer, progression to castration-resistant (CRPC) occurs despite castrate testosterone levels. Persistent androgen receptor (AR) signaling through amplification, mutation, or splice variants (AR-V7), as well as alternative pathways such as glucocorticoid receptor activation and lineage plasticity, drive resistance. Current therapeutic strategies include AR pathway inhibitors (enzalutamide, abiraterone), taxanes, PARP inhibitors for HRR mutations, and PSMA-targeted radioligand therapy (¹⁷⁷Lu-PSMA). Drug resistance remains a critical barrier to durable disease control in urologic oncology. Future management will rely increasingly on biomarker-driven, precision oncology approaches to overcome therapeutic resistance in both NMIBC and CRPC.

### Reprogramming the cold prostate tumor microenvironment as an actionable target for cancer therapy

#### *Andrea Alimonti* (Institute of Oncology Research, Bellinzona, Switzerland)

**Fig. 10:**
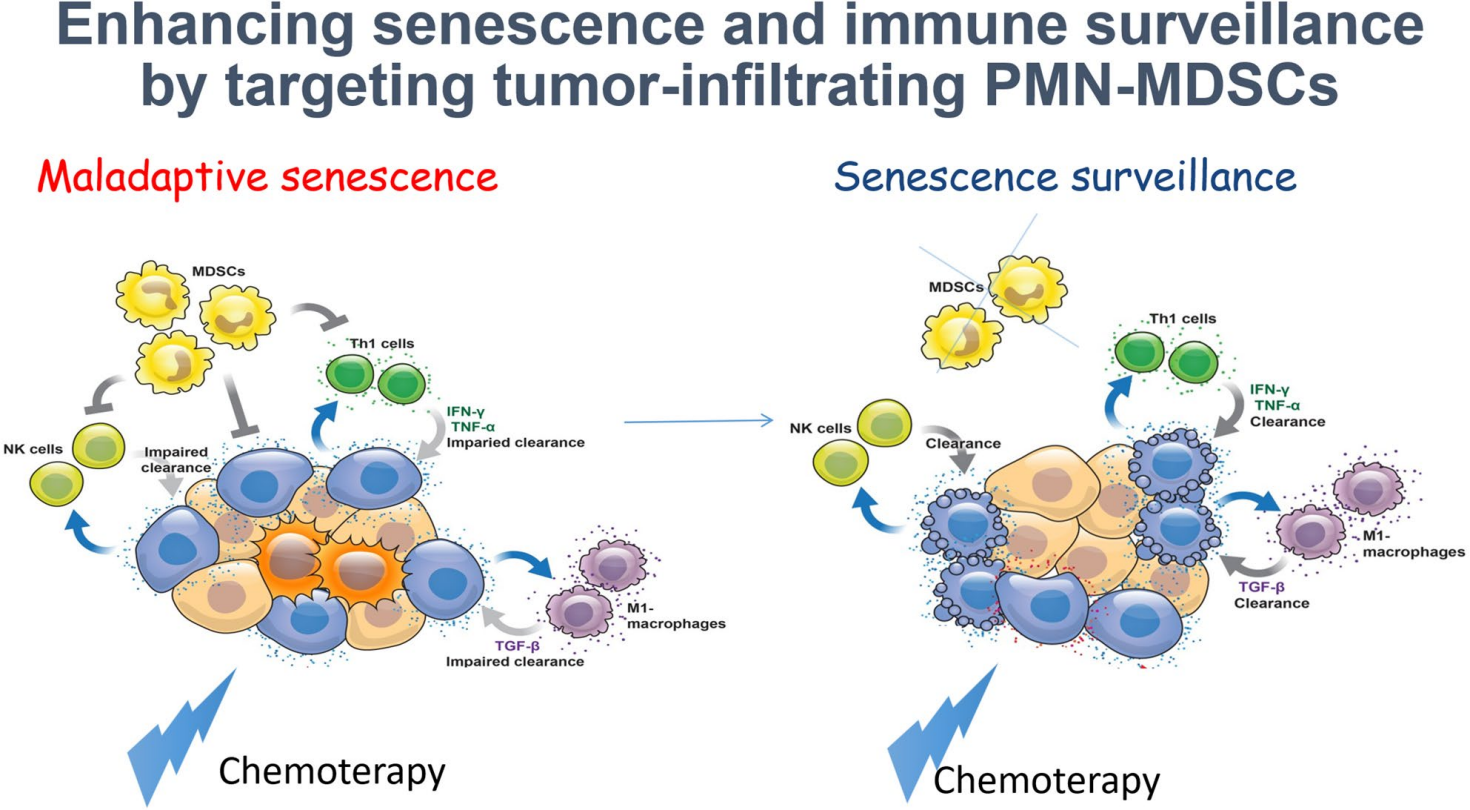
Adapted from Calcinotto A, Alimonti A. Aging tumour cells to cure cancer: “pro-senescence” therapy for cancer, Swiss Med Weekly [39].

Therapeutic failure in prostate cancer is driven not only by tumor-intrinsic mechanisms but also by a profoundly immunosuppressive tumor microenvironment that limits durable treatment responses. Therefore, strategies to reprogram the cold, immunosuppressed prostate tumor microenvironment (TME) into a therapeutically responsive state are needed. Cellular senescence plays a dual role in cancer: while it can suppress early tumor growth, senescent tumor cells can also promote metastasis and immunosuppression through the senescence-associated secretory phenotype (SASP) and the release of mitochondrial DNA [32, 33]. Myeloid cells, especially myeloid-derived suppressor cells (MDSCs), are key drivers of TME immunosuppression by blocking therapy-induced senescence and enabling tumor evolution, while tumor-associated macrophages (TAMs) can be reprogrammed via CXCR2 inhibition to induce TNFα-mediated senescence [34, 35]. Recent findings show that polymorphonuclear-MDSC (PMN-MDSCs) themselves can acquire a senescent-like state, becoming even more potently immunosuppressive and tumor-promoting. Eliminating these senescent-like myeloid cells with an HDAC inhibitor enhances therapy response [36]. Beyond this, PMN-MDSCs support resistance to androgen-deprivation therapy through IL-23 and coagulation Factor X [37, 38]. Emerging senolytic and SASP-modulating strategies targeting both tumor cells and immune cells show strong potential to limit metastasis and restore anti-tumor immunity. (Figure 10).

### Towards fast and accessible precision oncology

#### *Eytan Ruppin* (National Cancer Institute, Bethesda, USA)

I started my presentation by briefly reviewing my lab’s decade long work on developing a synthetic lethality approach for predicting patients’ response to therapy from tumor transcriptomics and \whole slide pathology images [Cell 2021, MED 2023, Nature Cancer 2024]. The main part of my talk focused on Path2Space, a new generic unpublished deep learning approach that predicts spatial gene expression directly from histopathology slides [40]. Applied to study breast cancer as an example, Path2Space robustly predicts the spatial gene expression of more than 4000 genes and accurately infers cell-type abundances in the tumor microenvironment (TME) based on the inferred ST data. Path2Space characterizes the TME of more than a 1000 breast cancer TCGA samples on an unprecedented scale and identifies three new spatially-grounded breast cancer patients subgroups with distinct survival rates. Closing a decade long circle, Path2Space- enables more accurate predictions of patients’ response to chemotherapy and trastuzumab directly from H&E slides than those obtained by existing established sequencing-based transcriptomics biomarkers. Path2Space thus offers a transformative approach to robustly delineate the tumors microenvironment directly from their histopathology slides. It facilitates the development of a new type of spatially-grounded biomarkers and lays the basis for a new generation of fast and low cost precision oncology biomarkers”.

#### Closing remarks

The 7th IRE Workshop on Translational Oncology provided an important forum for the exchange of ideas and for deepening our collective understanding of drug tolerance and resistance in cancer. Through 14 keynote lectures, a dedicated round table on advancing clinical trials, and 8 oral presentations by young investigators, the meeting showcased cutting-edge research spanning multiple dimensions of therapy resistance, from tumor-intrinsic plasticity to microenvironmental and spatially organized mechanisms. A unifying message was that resistance is not a static event but an adaptive trajectory shaped by dynamic interactions between cancer cells and their ecosystem. In this context, the future of oncology lies in intercepting these adaptive paths by integrating systems-level biology, spatially resolved analytics, and AI-powered diagnostics with rational, mechanism-based combination therapies. By fostering interdisciplinary dialogue and patient-inspired innovation, this workshop aimed to catalyze new collaborations and translational strategies, with the ultimate goal of converting transient therapeutic responses into durable clinical remissions.

## Data Availability

No datasets were generated or analysed during the current study.

## Abbreviations

BCG: Bacillus Calmette Guerin
ctDNA: Circulating Tumor DNA
CAF: Cancer-associated fibroblasts
TME: Tumor Microenvironment
NRF1: Nuclear Respiratory Factor 1
ASO: antisense oligonucleotides
MM: Multiple Myeloma
ICB: Immune checkpoint blockade
ET-1 R: ET-1 receptors
ECM: Extracellular Matrix
TAMs: Tumor-associated macrophages
PD: Patient-derived
MDSCs: Myeloid-derived suppressor cells
EC: Endometrial cancer
SASP: Senescence-associated secretory phenotype
NMIBC: Non muscle invasive bladder cancer
AR: Androgen receptor
CRPC: Castration-resistant prostate cancer
